# Macrolactins from Marine-Derived *Bacillus subtilis* B5 Bacteria as Inhibitors of Inducible Nitric Oxide and Cytokines Expression

**DOI:** 10.3390/md14110195

**Published:** 2016-10-26

**Authors:** Xia Yan, Yun-Xia Zhou, Xi-Xiang Tang, Xiu-Xiu Liu, Zhi-Wei Yi, Mei-Juan Fang, Zhen Wu, Fu-Quan Jiang, Ying-Kun Qiu

**Affiliations:** 1 Fujian Provincial Key Laboratory of Innovative Drug Target Research, School of Pharmaceutical Sciences, Xiamen University, South Xiang-An Road, Xiamen 361102, China; yanxia1201@126.com (X.Y.); yunxiazhouxmu@163.com (Y.-X.Z.); liuxiuxiu6@126.com (X.-X.L.); fangmj@xmu.edu.cn (M.-J.F.); wuzhen@xmu.edu.cn (Z.W.); 2 Key Laboratory of Marine Biogenetic Resources, Third Institute of Oceanography State Oceanic Administration, Xiamen 361005, China; tangxixiang@tio.org.cn (X.-X.T.); yizhiwei@tio.org.cn (Z.-W.Y.); 3 South China Sea Bio-Resource Exploitation and Utilization Collaborative Innovation Center, Xiamen 361005, China

**Keywords:** *Bacillus subtilis* B5, 7,13-epoxyl-macrolactin A, macrolactins, anti-inflammatory

## Abstract

In order to find new natural products with anti-inflammatory activity, chemical investigation of a 3000-meter deep-sea sediment derived bacteria *Bacillus subtilis* B5 was carried out. A new macrolactin derivative was isolated and identified as 7,13-epoxyl-macrolactin A (**1**). Owing to the existence of the epoxy ring, **1** exhibited a significant inhibitory effect on the expression of inducible nitric oxide and cytokines, compared with previously isolated known macrolactins (**2**–**5**). Real-time Polymerase Chain Reaction (PCR) analysis showed that the new compound significantly inhibited the mRNA expressions of inducible nitric oxide synthase (iNOS), interleukin-1β (IL-1β), and interleukin-6 (IL-6) in lipopolysaccharide (LPS)-stimulated RAW 264.7 macrophages. Reverse transcription-PCR analysis demonstrated that the new compound reduced the mRNA expression level of IL-1β in a concentration-dependent manner.

## 1. Introduction

The process of inflammation is the result of immune system activation which coordinates the normal defense mechanism of our body in response to microbial infection. Uncontrolled inflammation is believed to play crucial roles in the pathogenesis of various diseases, such as cardiovascular diseases [1], inflammatory bowel disease [2], cancer [3], diabetes [4], asthma [5], and Alzheimer’s disease [6]. During the inflammatory process, biochemical parameters, such as expression of inducible nitric oxide synthase (iNOS), cyclooxygenase-2 (COX-2), and 5-lipoxygenase [7,8], and levels of tumor necrosis factor-α (TNF-α), interferon γ (IFN-γ), interleukin-1 (IL-1), and interleukin-6 [9,10] are overexpressed during inflammation. Thus, inhibition of the production of these inflammatory mediators is an important target in the treatment of inflammatory diseases [11]. 

Several types of drugs are used to treat inflammatory disorders, such as biological, steroidal, and nonsteroidal anti-inflammatory drugs. However, they cause adverse side effects, and biological treatment is expensive. Natural products are alternatives to these drugs and offer hope for discovering bioactive lead compounds that may be developed into drugs for treatment of inflammatory disorders [12]. Plenty of unique marine natural products and their derivatives, such as sesquiterpenoid, polysaccharide, steroid/sterol and alkaloid, are found to manifest an anti-inflammatory action [13,14,15,16,17,18].

**Figure 1:**
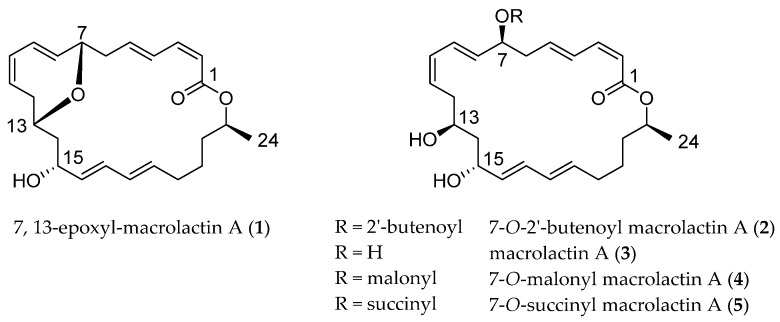
Structures of compounds **1**–**5** isolated from an extract of *Bacillus subtilis B5*.

Macrolactins, an important 24-membered macrolactones, are mainly produced by *Bacillus* [19,20,21,22]. In our previous study [19], a new macrolactin (**2**) and three known ones (**3**–**5**), were isolated from *Bacillus subtilis B5*, a bacteria derived from the 3000-meter deep-sea sediment of the Pacific Ocean. 7-*O*-2′*E*-Butenoyl macrolactin A (**2**) exhibited antifungal activity against tea pathogenic fungi *Pestalotiopsis theae* and *Colletotrichum gloeosporioides*. Continuous investigations on the chemical ingredients of *B. subtilis* B5 led to the isolation of another new macrolactin, 7,13-epoxyl-macrolactin A (**1**) (Figure 1). Studies on the anti-inflammatory activity of these macrolactins revealed that the new compound (**1**) exhibited potent activity, owing to the existence of an epoxy ring. The 3-(4,5-dimethylthiazol-2-yl)-5-(3-carboxymethoxyphenyl)-2-(4-sulfonyl)-2*H*-tetrazolium (MTS) assay demonstrated that these macrolactins did not exhibit obvious cytotoxic effect at the employed concentrations (1–40 μM) on RAW 264.7 cells. Compound **1** significantly inhibited the mRNA expressions of iNOS, IL-1β, and IL-6, while other known macrolactins showed fewer inhibitory effects in LPS-stimulated RAW 264.7 macrophages. Additionally, reverse transcription-PCR analysis demonstrated that the new compound inhibited the mRNA expression level of IL-1β in a concentration-dependent manner.

## 2. Results

### 2.1. Structural Identification of 7,13-Epoxyl-macrolactin A *(**1**)*

Compound **1** (7,13-epoxyl-macrolactin A) was isolated as a yellow amorphous powder. The molecular formula of C_24_H_32_O_4_, which gave 9 unsaturation degrees, was established by the HR-ESI-MS ion peak at *m*/*z* 407.2190 [M + Na] ^+^. The IR spectrum showed the presence of OH groups (3464 cm^−1^), olefinic protons (1450 cm^−1^) and carbonyls (1664 cm^−1^). The UV maximum absorption wave length at λ_max_ (log ε): 233 (3.88) nm indicating the presence of conjugated carbonyls. The ^1^H and ^13^C NMR spectra, including DEPT, clearly showed two carbonyl carbons and 12 olefinic methines belonging to 6 ethylenic bonds, in the *sp*^2^ low field region. The *sp*^3^ high field region showed the existence of a methyl, four oxygenated methines, and six methylenes.

**Figure 2:**
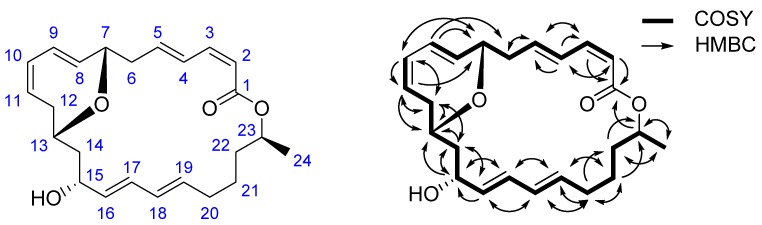
Structure and key ^1^H–^1^H COSY, HMBC correlations of **1**.

Most of the 1D NMR spectral data of **1** approach to those of macrolactin A (**3**), a typical macrolactin isolated from culture of a deep-sea marine bacterium [23], indicating the presence of a macrolactin nucleus in **1**. The molecular formula of **1**, when compared with that of **3**, found that an H_2_O unit was lost, suggesting the existence of an epoxy moiety. The ^13^C NMR signals assigned to C-8 and C-12 of macrolactin A were up-field shifted from δ 137.9 to δ 130.6 and from δ 36.0 to δ 31.3 respectively in **1**, indicating that the epoxy moiety was formed by the condensation between 7-OH and 13-OH. The chemical shifting effect was also observed in ^1^H NMR spectra, in which the H-7 signal of macrolactin A was down-field shifted from δ 4.19 to δ 4.56 in **1**, while 7-OH and 13-OH signal at δ 5.05 and δ 4.60 of macrolactin A were missing. Comprehensive ^1^H–^1^H COSY and HMBC analysis allowed the complete assignment of the proton and carbon signals for **1** (Table 1 and Figure 2). As a result, the structure of **1** was elucidated as 7,13-epoxyl-macrolactin A. It was a newly isolated compound produced by deep-sea sediment of the Pacific Ocean. Many isolated macrolactins including epoxyl-macrolactin that have been isolated [19,20,21,22] and all macrolactins contain three separate diene structure elements.

### 2.2. Anti-Inflamatory Activity of Macrolactins

#### 2.2.1. Cytotoxicity

**Figure 3:**
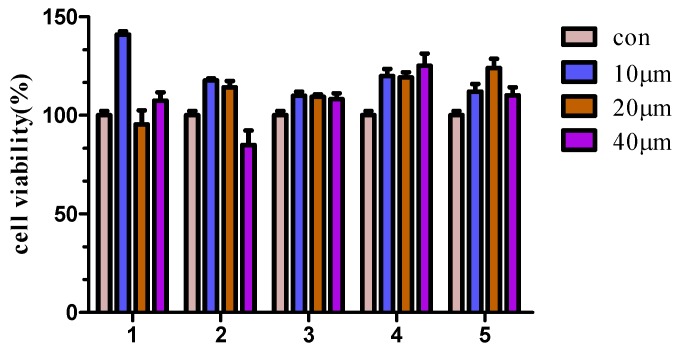
Compounds cytotoxicity at different concentrations on the viability of RAW 264.7 cells. Cells (1 × 10^4^ cells/well) plated on 96-well plates were treated with compounds **1**–**5** at concentrations of 10–40 μM at 37 °C for 24 h. Cytotoxicity was assessed by MTS assay. Values are expressed as mean ± SD, *n* = 5.

Cytotoxicity of compounds **1**–**5** in RAW 264.7 cells were tested by using the MTS assay. The result showed that compounds **1**–**5** did not exhibit obvious cytotoxic effect at the employed concentrations (10–40 μM) (Figure 3). Interestingly, Compound **1** can promote cell proliferation under the low concentration 10 μM. This may be because, in this case, it activates the cell survival. The detailed mechanism still needs further research.

#### 2.2.2. Inhibitory Effect of Compounds on LPS-Induced iNOS, IL-1β and IL-6 mRNA Expression

LPS can evoke innate immune response by stimulating the expression of several factors such as nitric oxide (NO) and pro-inflammatory cytokines, known to be involved in the immune response in macrophages. Compared with controls, upon LPS stimulation, macrophages strongly expressed the mRNA of iNOS, IL-1β and IL-6. Herein, compounds **1**–**5** were tested for in vitro anti-inflammatory activity and were found to suppress the mRNA expressions of iNOS, IL-1β and IL-6 in LPS-stimulated RAW 264.7 macrophages.

**Figure 4:**
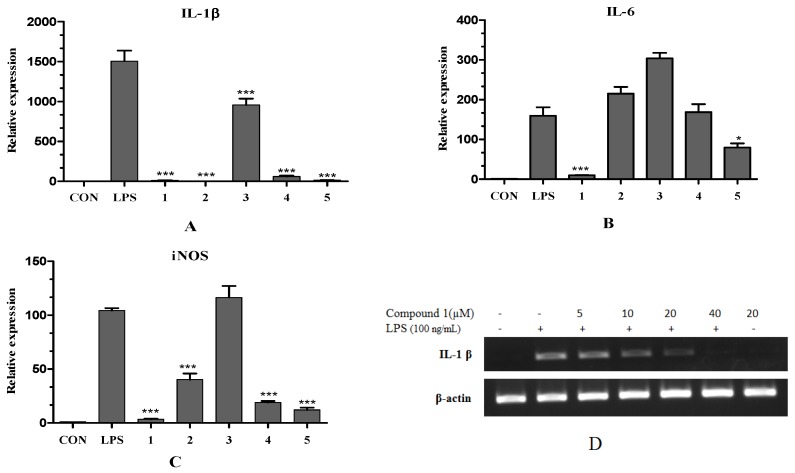
Effects of compounds **1**–**5** on the mRNA expression of LPS-induced interleukin-1β (IL-1β) (**A**), interleukin-6 (IL-6) (**B**) and nitric oxide synthase (iNOS) (**C**) of RAW 264.7 cells at the concentration of 40 μM by Real-Time Polymerase Chain Reaction Analysis. (**D**) Effects of **1** on IL-1β mRNA expressions at concentrations of 5–40 μM by Reverse Transcription-Polymerase Chain Reaction Analysis. The β-actin was used as a loading control. The cells in a six-well were pretreated with compounds for 1.5 h and then stimulated with LPS (100 ng/mL) for another 12 h. LPS-induced mRNA expression of iNOS and IL-6 was reduced by 96.6% and 93.8%, respectively, when cells were cotreated with compound **1**. Total RNAs were isolated from cells and analyzed. The *p* values were assessed by Student’s *t* test. A p value less than 0.05 was considered statistically significant (* *p* < 0.05; *** *p* < 0.001). (Data represent the mean ± SD of different experiments.

As shown in Figure 4A–C, pretreatment of LPS activated cells with compounds **1** and **5** resulted in significant reduction of the mRNA expression of IL-1β, IL-6 and iNOS. Compounds **2** and **4** reduced the production of IL-1β and iNOS but had little effect on the expression level of IL-6. Compound **3** only slightly reduced the mRNA expression of IL-1β.

Based on the above results, we further investigated the effects of **1** on IL-1β mRNA expressions in LPS-stimulated RAW 264.7 macrophages by Reverse Transcription-PCR analysis. As shown in Figure 4D, compound **1** inhibited IL-1β mRNA expression in a concentration-dependent manner, while the house-keeping β-actin mRNA expression was unchanged by compound **1** under the same condition.

## 3. Materials and Methods

### 3.1. General Experimental Procedures

The HR-ESI-MS analysis was performed on the Thermo Q-Exactive Orbitrap Mass spectrometer (Thermo Fisher Scientific Corporation, Waltham, MA, USA) equipped with electrospray ionization source (ESI). The preparative HPLC was performed with Varian binary gradient LC system (Varian Inc. Corporate, Santa Clara, CA, USA) containing two solvent deliver modules (PrepStar 218), a photodiode array detector (ProStar 335) and a fraction collector (ProStar 704), using an the preparative Cosmosil ODS column (250 mm × 20.0 mm i.d., 5 μm, Cosmosil, Nakalai Tesque Co. Ltd., Kyoto, Japan). UV spectra were recorded on a Shimadzu UV-260 spectrometer (Shimadzu Corporation, Tokyo, Japan). IR spectra were determined on a Perkin-Elmer 683 infrared spectrometer (PerkinElmer, Inc., Waltham, MA, USA) in KBr pellets. Optical rotations were measured using a JASCO P-200 polarimeter (JASCO Corporation, Tokyo, Japan) with a 5-cm cell. The NMR spectra were taken with TMS as the internal standard on a Brucker Avance III 600 FT NMR spectrometer (Bruker Corporation, Billerica, MA, USA). Column chromatography was performed on silica gel (Yantai Chemical Industry Research Institute, Yantai, China) and Cosmosil 75 C_18_-OPN (75 μm, Nakalai Tesque Co. Ltd., Kyoto, Japan).

### 3.2. Bacteria and Fermentation

The bacterial strain B5 was isolated from deep-sea sediments which were collected at water depths of 3000 m Pacific Ocean by the Third Institute of Oceanography of China, and was identified as *Bacillus subtilis B5* by complete 16S rRNA gene sequence. This bacterium was cultivated on 160 L scale using 250 mL shake flasks containing 100 mL of the seed medium (tryptone 1%, yeast extract 0.5%, NaCl 1%, pH 7.4) cultured at 37 °C for 18 h at 200 rpm. One percent of the seed culture was inoculated into 100 L fermentors containing 80 L of fermentation medium consisting of soluble starch 0.5%, yeast extract 1%, K_2_HPO_4_ 1%, NaNO_3_ 0.5%, pH 6.8–7.0 for 2 days at the same condition mentioned above, and fermentations were conducted twice.

### 3.3. Extraction and Isolation

HPD-100 macroporous adsorption resin (Cangzhou Bonchem Co., Ltd., Changzhou, China) was used to deal with the fermentation broth (160 L). After being eluted with water, the 95% ethanol eluents were collected. After removal of ethanol under vacuum, the water suspension (1.5 L) was extracted with ethyl acetate (*v*/*v* 1:1) three times. The organic phase was evaporated under reduced pressure to afford a residue (76 g). The residue was divided into 11 fractions (Fr. 1~11) over silica gel column eluted with petroleum ether-ethyl acetate (*v*/*v*) (50:1; 20:1; 10:1; 5:1; 2:1; 1:1) and chloroform-methyl alcohol (*v*/*v*) (50:1; 20:1; 10:1; 5;1; 2:1). Fr. 5 (1.2 g) was subjected to ODS chromatography and eluted with MeOH–H_2_O (30%–100%) to give a subfraction Fr. 5.6. Then Fr. 5.6 was purified by preparative reversed-phase HPLC using a C_18_ column and isocratic eluted with acetonitrile-H_2_O (62:38) to obtain compound **1** (9 mg).

7,13-epoxyl-macrolactin A (**1**): Amorphous white powder; mp: 92–96 °C; ${\left[\alpha \right]}_{D}^{25}$ −34° (*c* = 0.1, MeOH), IR (KBr) (ν_max_): 3465, 3268, 1664, 1450 cm^−1^. UV (MeOH) λ_max_ (log *ε*): 233 (3.88) nm. ^13^C NMR (125 MHz, DMSO-*d_6_*) and ^1^H NMR (600 MHz, DMSO-*d_6_*) spectral data were listed in Table 1; HR-ESI-MS: *m*/*z* 407.2190 (calcd. for C_16_H_11_N_2_O_3_BrNa, 407.2193).

### 3.4. Cell Cultivation

The murine macrophage cell line RAW 264.7 was purchased from ATCC (Rockville, MD, USA). RAW 264.7 cells were cultured in Dulbecco’s modified Eagle’s medium (DMEM, Hyclone, Logan, UT, USA) supplemented with 1% penicillin-streptomycin (100 U/mL penicillin and 100 μg/mL streptomycin in 0.85% NaCl) and 10% of fetal bovine serum (FBS, Hyclone, Logan, UT, USA). The cells were incubated in a humidified atmosphere of 5% CO_2_ at 37 °C.

### 3.5. Cytotoxicity Assay

The cytotoxicity of compounds **1**–**5** were assessed by CellTiter 96^®^ AQueous One Solution Cell Proliferation Assay (Promega Corporation, Madison, WI, USA). RAW 264.7 cells (1 × 10^4^ cells/well) plated on 96-well plates were treated with different concentrations of samples (1–40 μM in DMSO) for 24 at 37 °C in 5% CO_2_. With a pipet, we put 20 μL of CellTiter 96^®^ AQueous One Solution Reagent (MTS) into each well of the 96-well assay plate containing the samples in 100 μL of culture medium. Then, the plate was incubated at 37 °C for 3 h in a humidified, 5% CO_2_ atmosphere and then the absorbance was recorded at 490 nm with a 96-well plate Microplate reader MK3 (Thermo Fisher Scientific Corporation, Waltham, MA, USA). The optical density of formazan formed in control cells (without treatment with samples) was taken as 100% viability.

### 3.6. Total RNA Isolation

RAW 264.7 cells were plated onto 6-well plates at a density of 1 × 10^6^ cells/well and incubated with compounds at the indicated concentration for 1.5 h prior to LPS (100 ng/mL) stimulation. After 12 h, cells were lysed and total RNA extraction was performed by using Trizol reagent (Life Technologies, Carlsbad, MA, USA). Cells were homogenized in 200 μL of Trizol reagent, and then samples were left to rest at room temperature for 5 min. After that, 40 μL of chloroform was added and the tubes were vigorously shaken for 15 s and allowed to rest at room temperature for 5 min. Tubes were then centrifuged at 12,000× *g* (Eppendorf Centrifuge 5424 R, Eppendorf Instruments, Hamburg, Germany) 4 °C for 15 min. The aqueous phase was transferred to a new tube. Isopropyl alcohol (100 μL) was added to the aqueous phase: the tube was then gently mixed and incubated at room temperature for 10 min. After incubation, samples were centrifuged at 12,000× *g*, 4 °C for 10 min. The supernatant was poured out and the pellet was washed by 200 μL of 75% ethanol and centrifuged at 12,000× *g*, 4 °C for 5 min. The washing step and centrifuge were repeated. The final supernatant was removed and the pellet was dried until it was colorless. Total RNA was then dissolved in 20 μL of DEPC H_2_O, incubated at 65 °C for 5 min, and stored at −80 °C until used.

### 3.7. Real-Time PCR Analysis of iNOS, IL-1β and IL-6 mRNA

The inhibitory effect of compounds **1**–**5** on the mRNA expression of LPS-induced IL-1β, IL-6 and iNOS of RAW 264.7 cells at the concentration of 40 μM in DMSO was analyzed by Real-Time PCR. The expression of mRNA transcripts of iNOS (forward: 5′-GTTCTCAGCCCAACAATACAAGA-3′, reverse: 5′-GTGGACGGGTCGATGTCAC-3′), IL-1β (forward: 5′-GAAATGCCACCTTTTGACAGTG-3′, reverse: 5′-TGGATGCTCTCATCAGGACAG-3′), IL-6 (forward: 5′-CCAGAGATACAAAGAAATGATGG-3′, reverse: 5′-ACTCCAGAAGACCAGAGGAAAT-3′) and β-actin (forward: 5′-GGCTGTATTCCCCTCCATCG-3′, reverse: 5′-CCAGTTGGTAACAATGCCATGT-3′) was determined by real-time RT-PCR. The cDNA was synthesized from total RNA using oligo (dT)_18_ primers. FastStart Universal SYBR Green Master (Rox, Roche, Basel, Switzerland) and Stepone Real-Time PCR Detection System (Applied Biosystems, Foster, CA, USA) were used for Real-time PCR analysis. Levels of all mRNAs were normalized to that of GAPDH mRNA.

### 3.8. Reverse Transcription-PCR Analysis of IL-1β mRNA

The inhibitory effect of compound **1** at the indicated concentrations on IL-1β mRNA expressions was determined by Reverse Transcription-PCR. Primers used were as follows: IL-1β (forward: 5′-GGGCCTCAAAGGAAAGAATC-3′, reverse: 5′-TACCAGTTGGGGAACTCTGC-3′), β-actin (forward: 5′-CCACAGCTGAGAGGGAAATC-3′, reverse: 5′-AAGGAAGGCTGGAAAAGAGC-3′). The total RNA was converted to cDNA and analyzed. PCR amplifications were performed on an Eppendorf traditional PCR machine (Eppendorf Instruments, Hamburg, Germany). The cDNA was amplified with 30 cycles of 95 °C for 45 s, 60 °C for 15 s, and 72 °C for 45 s. After amplification, the PCR products were separated on 1.5% agarose gel for 30 min at 100 V. 

## 4. Conclusions

A new macrolactins derivatives 7,13-epoxyl-macrolactin A (**1**), was isolated from a marine-derived bacterial strain *Bacillus subtilis B5*. Evaluation of its anti-inflammatory effect indicated that the new compound inhibited the expression of proinflammatory cytokines IL-1β, IL-6 and iNOS induced by LPS in RAW 264.7 macrophages, similar to the effect of 7-*O*-succinyl macrolactin A (**5**). 7-*O*-2′*E*-butenoyl macrolactin A (**2**) and 7-*O*-malonyl macrolactin A (**4**) reduced the production of IL-1β and iNOS but had no effect the level of IL-6. Macrolactin A (**3**) only slightly reduced the production of IL-1β. Additionally, **1** inhibited IL-1β mRNA expressions in in a concentration-dependent manner. Based on the anti-inflammatory effects of compounds **1**–**5** in LPS-stimulated RAW 264.7 macrophages, it could be suggested that different substituents at the position 7 might be key structural features for the anti-inflammatory activity. The presence of the epoxy moiety was special in the structure of the new compound and might greatly affect the anti-inflammatory activity.

## Figures and Tables

**Table 1:** ^1^H, ^13^C NMR data and ^1^H–^1^H COSY, HMBC correlations of compound **1**.

Position	δ_H_ (*J* in Hz)	δ_C_, Multiple		^1^H–^1^H COSY	HMBC
1		166.0	C		
2	5.59 br.d (11.4)	117.8	CH	H-3	C-1,4
3	6.69 br.t (11.4)	143.4	CH	H-2,4	C-1,5
4	7.11 br.dd (14.7, 11.7)	128.1	CH	H-3,5	C-2,3,5
5	6.17 dt (14.9, 7.4)	142.1	CH	H-4,6	C-3,6
6	2.91 m	36.0	CH_2_	H-5	C-5
7	4.56 br.s	72.2	CH	H-8	n. o. ^a^
8	5.48 overlapped	130.6	CH	H-7,9	C-6
9	5.54 m	129.7	CH	H-8	C-5,6,7
10	5.71 br.d (10.5)	128.4	CH	H-11	C-7
11	5.81 m	125.2	CH	H-10,12	C-12
12	1.98 m and 1.87 m	31.3	CH_2_	H-11,13	C-8,10,13,14
13	3.50 overlapped	65.0	CH	H-12,14	n. o.
14	1.68 m and 1.61 m	44.1	CH_2_	H-13,15	C-15,16
15	3.93 br.s	69.4	CH	H-14,16	C-14
16	5.48 overlapped	135.3	CH	H-15,17	C-14,15
17	6.06 br.dd (14.9, 10.5)	130.1	CH	H-16,18	C-15
18	5.97 br.dd (14.9, 10.6)	130.3	CH	H-17,19	C-16,20
19	5.67 m	133.9	CH	H-18,20	C-20,21
20	2.13 m and 2.00 m	32.1	CH_2_	H-19,21	C-19,21,22
21	1.44 m	25.4	CH_2_	H-20,22	C-19,20,22
22	1.59 m	35.3	CH_2_	H-21,23	C-20,21,24
23	4.96 m	70.8	CH	H-22,24	C-1,21,22,24
24	1.22 d (6.2)	20.4	CH_3_	H-23	C-22,23
15-OH	4.65 br.s				

^a^ n. o. is not observed.
